# Brain expansion promoted by polycomb-mediated anterior enhancement of a neural stem cell proliferation program

**DOI:** 10.1371/journal.pbio.3000163

**Published:** 2019-02-26

**Authors:** Shahrzad Bahrampour, Carolin Jonsson, Stefan Thor

**Affiliations:** 1 Department of Clinical and Experimental Medicine, Linkoping University, Linkoping, Sweden; 2 School of Biomedical Sciences, University of Queensland, St. Lucia, Queensland, Australia; New York University, UNITED STATES

## Abstract

During central nervous system (CNS) development, genetic programs establish neural stem cells and drive both stem and daughter cell proliferation. However, the prominent anterior expansion of the CNS implies anterior–posterior (A–P) modulation of these programs. In *Drosophila*, a set of neural stem cell factors acts along the entire A–P axis to establish neural stem cells. Brain expansion results from enhanced stem and daughter cell proliferation, promoted by a Polycomb Group (PcG)->Homeobox (Hox) homeotic network. But how does PcG->Hox modulate neural-stem-cell–factor activity along the A–P axis? We find that the PcG->Hox network creates an A–P expression gradient of neural stem cell factors, thereby driving a gradient of proliferation. PcG mutants can be rescued by misexpression of the neural stem cell factors or by mutation of one single Hox gene. Hence, brain expansion results from anterior enhancement of core neural-stem-cell–factor expression, mediated by PcG repression of brain Hox expression.

## Abstract 2

A study in fruit flies shows that the anterior expansion of the central nervous system, to form the brain, is driven by Polycomb-mediated repression of Hox genes, resulting in anterior enhancement of a neural stem cell program.

## Author summary

The central nervous system displays a pronounced anterior expansion that forms the brain. In the fruit fly *Drosophila melanogaster*, this expansion is driven by enhanced anterior cell proliferation. Recent studies reveal that cell proliferation in the brain is promoted by the Polycomb Group Complex, a key epigenetic complex. During development of the central nervous system, the Polycomb Group Complex acts to exclude Hox homeotic gene expression from the brain, thereby rendering the brain a Hox-free zone. Hox genes act in an antiproliferative manner, which explains the hyperproliferation observed in the brain, as well as the gradient of proliferation along the anterior–posterior axis of the central nervous system. Here, we find that Hox genes act by repressing a common neural stem cell proliferation program in more posterior regions, resulting in an anterior–posterior gradient of “stemness.” Hence, elevated anterior proliferation is promoted by the Polycomb Group Complex acting to keep the brain free of negative Hox input, thereby ensuring elevated expression of neural stem cell factors in the brain. Strikingly, mutants of the Polycomb Group Complex can be rescued by mutation of one single Hox gene, demonstrating that the primary role of the Polycomb Group Complex is indeed Hox repression. This study advances our understanding of how neural stem cell programs operate at different axial levels of the central nervous system and may have implications also for stem cell and organoid biology.

## Introduction

During central nervous system (CNS) development, neural progenitor cells undergo repetitive rounds of asymmetric cell divisions, renewing themselves and generating daughter cells. After a certain number of cell divisions, neural progenitors subsequently exit the cell cycle. Daughter cells, in turn, may directly differentiate into neurons or glia or divide one or many times to expand any given lineage. Thus, lineage size depends upon two fundamental proliferation decisions: how many times should each progenitor divide, and how many times should its daughter cells divide? Studies have revealed profound differences in both progenitor and daughter cell proliferation behavior when comparing along the anterior–posterior (A–P) axis and over developmental time [1–7]. Such differences can result in striking alterations in lineage size when comparing between different progenitors, from a few to several hundred cells generated from one progenitor [8–13]. Modulation of lineage size can influence CNS regional size and contribute to the prominent anterior expansion of the CNS [3, 7, 14]. However, how neural progenitor and daughter cell proliferation is modified along the A–P axis and over developmental time to thereby result in changes in lineage and ultimately regional CNS size is still poorly understood.

**Fig 1:**
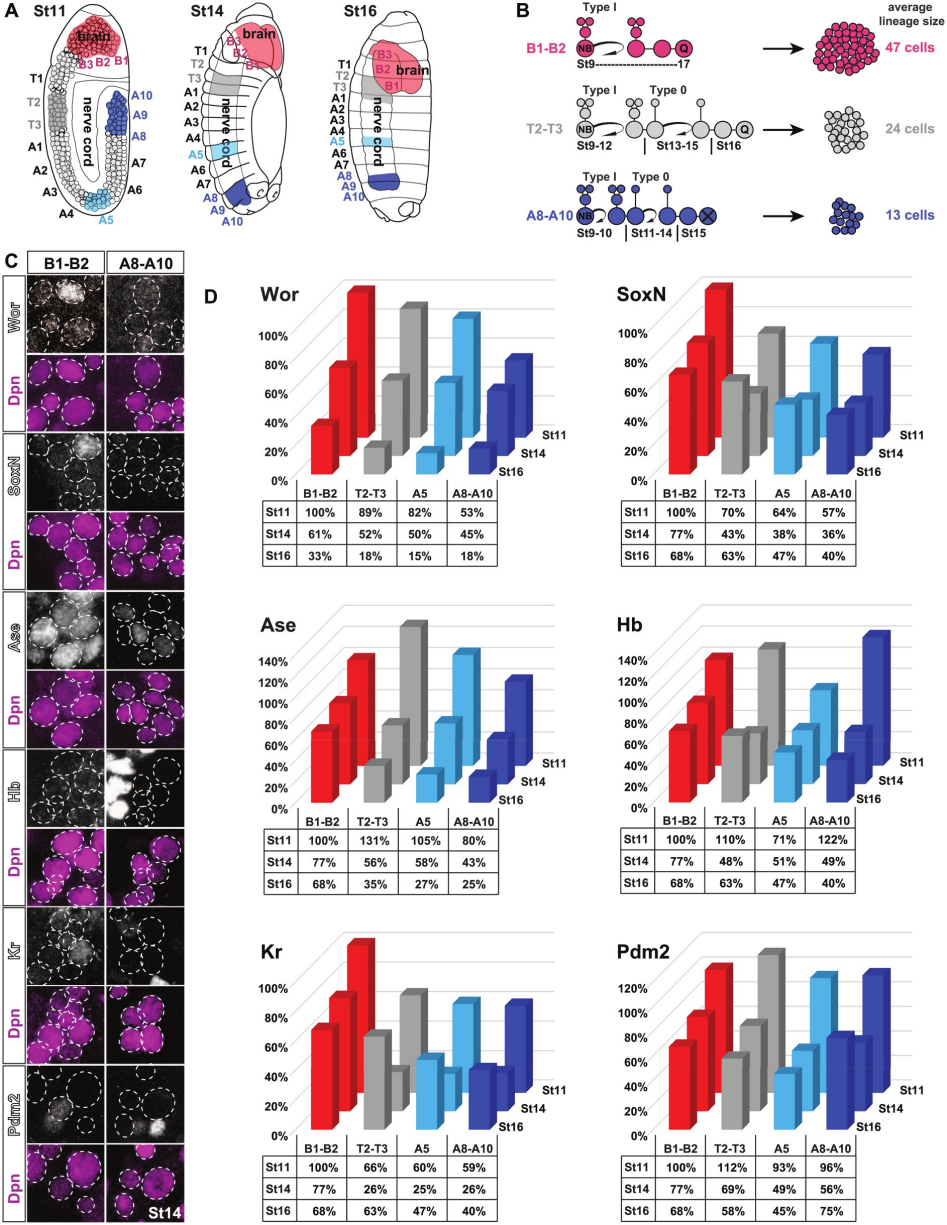
Expression of EFs is higher in brain. (A) Schematic of *Drosophila* embryos, showing early delaminating NBs in the neuroectoderm at St11 and development of the CNS at St14 and St16. (B) The majority of NB lineages initially progress in the Type I mode, generating daughter cells that divide once. In the nerve cord, at a programmed stage of lineage progression, many NBs switch to generating neurons directly (Type 0 mode). After a stereotyped number of divisions, different for each NB lineage, NBs exit the cell cycle, and most brain and thoracic NBs enter quiescence, while most abdominal NBs undergo apoptosis. The apparent lack of the Type I->0 switch in the brain and the longer phase of NB proliferation therein, as well as a gradient of the switch and NB exit in the nerve cord, results in profound differences in the average lineage sizes generated at different A–P levels. (C) Staining for pan-neural and temporal factors in brain (B1–B2) and posterior nerve cord (A8–A10) segments at St14. (D) Graphs summarizing the expression of EF in the different regions of the CNS, at the different stages examined (see S1 Fig for details). The numerical data underlying this figure are included in S1 Data. Genotypes: (C–D) *OregonR*. A–P, anterior–posterior; Ase, Asense; CNS, central nervous system; Dpn, Deadpan; EF, Early Factor; Hb, Hunchback; Kr, Kruppel; NB, neuroblast; Pdm, POU domain; SoxN, SoxNeuro; St, Stage; Wor, Worniou.

The developing *Drosophila* CNS is a powerful model system for addressing these issues [15]. The *Drosophila* CNS, subdivided into the brain and the nerve cord, is formed by approximately 1,200 bilateral neuroblasts (NBs) and a smaller number of midline NBs, all of which form in the neuroectoderm during early-to-mid embryogenesis (Fig 1A) [10, 12, 13, 16–18]. After delaminating from the neuroectoderm, NBs undergo a series of asymmetric cell divisions, renewing themselves while budding off daughter cells with reduced proliferative potential. The majority of NBs initially generate daughters that divide once to make two neurons/glia, denoted Type I proliferation mode [9]. Subsequently, after a stereotyped number of NB divisions, many NBs in the nerve cord switch to budding off daughter cells that differentiate directly, denoted Type 0 mode, and hence they undergo a programmed Type I->0 daughter cell proliferation switch [19]. Finally, the majority of NBs in the brain and nerve cord appear to exit the cell cycle after a programmed number of divisions, unique to each NB subtype (Fig 1B). The Type I->0 daughter cell proliferation switch and NB exit occurs in a graded fashion along the CNS, and the majority of brain NBs appear to stay in the Type I mode throughout embryogenesis. This results in striking differences in the average lineage size along the CNS A–P axis (Fig 1B) [3, 7].

In the nerve cord, NB cell-cycle exit and the Type I->0 daughter cell proliferation switch are under the control of an elaborate program of proliferation “drivers” and “stoppers.” These include all members of the so-called temporal gene cascade: Hb->Kr->Nubbin/POU domain 2 (Nub/Pdm2; collectively referred to as Pdm)->Castor (Cas)->Grainy head (Grh), playing out in the majority of embryonic NBs (reviewed in [20]). The temporal cascade controls NB competence and governs the generation of specific glia and neurons at distinct stages of lineage progression [20]. However, the temporal genes also control proliferation, with the early temporal factors Hb, Kr, and Pdm acting in a pro-proliferative manner and the late temporal factors Cas and Grh acting in an antiproliferative manner [19, 21]. In addition to the temporal factors, the pan-NB factors of the sex determining region Y-box B (SoxB) family (SoxNeuro [SoxN] and Dichaete [D]), the Snail family (Snail, Worniou [Wor], and Escargot), and the basic helix-loop-helix (bHLH) factor Asense (Ase) also control NB generation and development [21–29] and play key roles in driving proliferation [21, 23, 30]. While most, if not all, NBs express one or several of each pan-NB factor family member [23–28, 30–33], detailed expression analysis revealed that their expression is down-regulated during embryonic neurogenesis [21]. Herein, we refer collectively to the three early temporal factors and the pan-NB factors as Early Factors (EFs). The EFs are necessary and partly sufficient for several aspects of the NB program, including asymmetric cell division [23, 30], the Type I daughter cell proliferation mode, and continuing NB proliferation [21]. By a complex regulatory temporal interplay, EFs are gradually and precisely replaced by the late factors Cas and Grh, which triggers the Type I->0 daughter cell proliferation switch and, ultimately, NB exit [19, 21]. In addition to this temporal program, there is critical control of NB and daughter cell proliferation mediated by A–P cues [4, 5]. These cues are provided by the Hox homeotic genes, which are activated late in nerve cord NBs and act with Cas and Grh to trigger the Type I->0 switch and NB exit [3, 19, 34]. Anteriorly, the Polycomb Group (PcG) complex, in particular Polycomb Repressor Complex 2 (PRC2), acts to keep the brain free of Hox homeotic gene expression, thereby preventing the Type I->0 daughter cell proliferation switch and promoting an extended phase of NB proliferation [7]. However, these studies raise the question of how the PcG->Hox program modulates EF activity along the A–P axis to promote the gradient in NB and daughter cell proliferation.

We find that EF expression is elevated and extended in brain NBs when compared to the nerve cord. EFs are necessary for brain proliferation, and EF co-misexpression overrides Type I->0 switches and NB exit, both in the nerve cord and brain, resulting in a striking increase in overall CNS size. Elevated EF levels in the brain and graded EF expression along the nerve cord are both gated by the PcG->Hox network. Strikingly, the effects of PRC2 mutation—including the nondetectable levels of Histone 3 K27 trimethylation (H3K27me3), the “invasion” of Hox gene expression into the brain, the reduction of EF expression, and the accompanied reduction of proliferation—can be rescued by mutation in the Hox gene *Abd-B* or by transgenic expression of EFs. These results demonstrate that the PcG->Hox network modulates a temporal neural stem cell program along the A–P axis, thereby allowing for the wedge-like development of the CNS, with its prominent anterior expansion.

## Results

### Gradient of EF expression in the CNS

We previously focused on thoracic segments T2–T3, finding that the three pan-neural factors Wor, SoxN, and Ase, as well as the three early temporal factors Hb, Kr, and Pdm, are expressed in early NBs but down-regulated during lineage progression [21]. This down-regulation is necessary for the Type I->0 daughter cell proliferation switch and the final NB cell-cycle exit. To address whether the expression levels of these six factors, collectively referred to herein as EFs, correlate with the gradient of the Type I->0 switch and NB exit observed in the developing *Drosophila* CNS, we analyzed EF expression levels in NBs in the brain (B1–B2) and thorax (T2–T3), as well as two abdominal regions (A5 and A8–A10) (Fig 1A and 1C). We analyzed three stages: Stage (St)11, St14, and St16, chosen because they represent the gradual accentuation of A–P proliferation differences [3, 7, 19, 21]. Specifically, at St11, there are only minor differences in NB and daughter cell proliferation along the A–P axis, with most NBs and daughter cells proliferating (Type I). At St14, many NBs in the A8–A10 region have stopped dividing, daughter cells are mostly nondividing (Type 0), and many T2–T3 NBs have undergone the Type I->0 switch. At St16, proliferation of NBs and daughter cells in both abdomen and thorax have largely ceased, while many NBs and daughter cells in the B1–B2 region continue dividing.

At St11, as anticipated from the minor differences in proliferation along the A–P axis at this stage, we observed that only four out of the six EFs showed a higher expression level in B1–B2 when compared to A8–A10 (S1A Fig). Wor, SoxN, and Kr furthermore displayed higher levels in B1–B2 also when compared to T2–T3 and/or A5. In contrast, Ase, Hb, and Pdm2 showed a more complex picture, with Ase and Pdm2 being highest in T2–T3, and Hb highest in A8–A10 (S1A Fig). At St14, in line with the accentuated A–P proliferation differences, levels of all six EFs were significantly higher in B1–B2 than A5 and A8–A10, with five of six also significantly higher in B1–B2 than T2–T3 (S1B Fig). At St16, similarly, all six EFs were significantly higher in B1–B2 when compared to one or several nerve cord regions (S1C Fig). A peculiar exception observed pertains to Pdm2 levels, which showed a gradient from B1–B2 to T2–T3 and on to A5 but was then elevated in A8–A10 (S1C Fig). To address EF expression over time, we analyzed St11, St14, and St16 on the same slide and focused on the levels in B1–B2. This revealed that all six EFs were significantly down-regulated between St11 versus St14 and/or St16 and St14 versus St16 (S1D Fig). Kr differed between St11 and St16 and St14 and St16 but not between St11 and St14 (S1D Fig). Setting the expression levels at St11 in B1–B2 as 100% and normalizing against this allowed for the generation of 3D graphs that illustrate the EF expression landscape over time and along the A–P axis (Fig 1D). The most salient feature was that during early stages, EFs were robustly expressed in NBs at all axial levels. As development progressed and A–P differences in NB and daughter proliferation play out, EFs were generally down-regulated in a graded manner in the nerve cord but maintained for longer in the brain.

### Early mutants show reduced NB and daughter cell proliferation

Previous studies focused on the role of EFs in the thoracic segments T2–T3, revealing that they promote NB and daughter cell proliferation [21]. To determine if they play similar roles throughout the CNS, we analyzed brain segments B1–B2, as well as the nerve cord abdominal segments A1, A5, and A8–A10.

To analyze NB and daughter cell proliferation in the developing CNS, we used Prospero (Pros), Deadpan (Dpn), and phosphorylated Ser10 on Histone-H3 (PH3) as markers. This approach relies on the fact that dividing NBs are PH3+, Pros-cortical asymmetric, and Dpn+, while dividing daughter cells are PH3+, Pros cytoplasmic, and Dpn negative (S2A Fig) [19]. We analyzed proliferation at St14 in the EF mutants *wor*, *SoxN*, *ase*, *hb*, *Kr*, and *nub*,*pdm2* (the function of the adjacent *nub* and *pdm2* genes was addressed simultaneously by using a genomic deletion for both genes, referred to as *nub*,*pdm2*). We observed significantly reduced NB proliferation in B1–B2 in all mutants except *SoxN* (S2B–S2D Fig). In abdominal segments, all mutants showed reduced proliferation, with the exception of *SoxN* in A1 and *nub*,*pdm2* in all segments (S2D Fig).

With respect to daughter cell proliferation, there was significantly reduced proliferation in B1–B2 in all mutants except *nub*,*pdm2* (S2E Fig). The effects were generally weaker in the abdominal segments, with only *wor* and *ase* showing significant effects in A1, none of the genes showing effects in A5, and with *wor*, *ase*, and *Kr* showing effects in A8–A10 (S2E Fig). The role of *hb* could not be addressed in A8–A10 because of a loss of these segments in the compound mutant used herein.

We conclude that all of the six EFs are important for NB and/or daughter cell proliferation in the brain. With the exception of *nub*,*pdm2*, they are also important for NB proliferation in the abdominal segments, while their involvement in abdominal daughter cell proliferation is less pronounced.

### EF overexpression blocks daughter cell switches and NB exit

We previously demonstrated that the brain is “hyperproliferative” when compared to the nerve cord, displaying an apparent lack of the Type I->0 daughter cell proliferation switch and an extended NB proliferation phase [7]. Herein, we observed elevated and extended EF expression in the brain and the importance of EFs for NB and daughter cell proliferation in the brain. In combination, these findings suggested that elevated and extended EF expression could be a contributing factor to the enhanced proliferation normally observed in the brain. To address this notion, we co-misexpressed all six factors (upstream activating sequence [*UAS*]*-6xEF*) during CNS development, using the *inscuteable-Galactose4* (*insc-Gal4*) driver (S3A–S3Z Fig).

**Fig 2:**
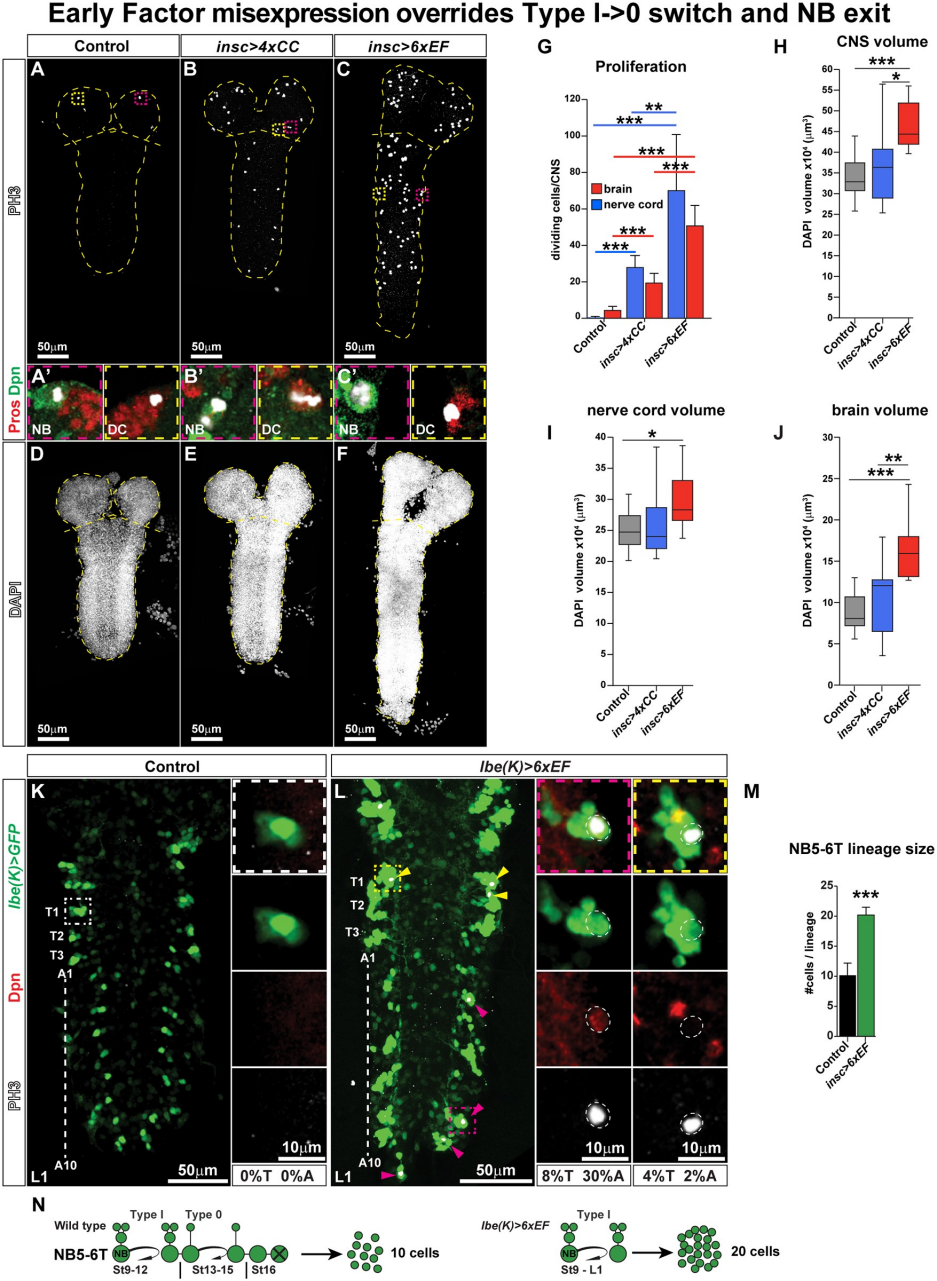
EF co-misexpression overrides the Type I->0 switch and NB exit. (A–F) Whole CNS, AFT stage, stained for PH3 (A–C) or DAPI (D–F). (A) In control, a small number of dividing cells is observed in the brain and an occasional division in the nerve cord. (B) In *insc-Gal4/UAS-4xCC*, aberrant cell divisions are observed in both the brain and nerve cord. (C) In *insc-Gal4/UAS-6xEF*, aberrant divisions are even more frequent. (A′–C′) Close-up of dividing NBs and daughter cells, identified by PH3, Dpn, and Pros. (G) Quantification of proliferation in the brain and nerve cord. Both *UAS-4xCC* and *UAS-6xEF* CNSs show significantly more proliferation than control, and *UAS-6xEF* significantly more than *UAS-4xCC* (*p ≤ 0.05, **p ≤ 0.01, ***p ≤ 0.001, Student two-tailed *t* test; *n* = 10 CNSs; ±SD). (H–J) Quantification of DAPI cell volume, in the entire CNS (H), the nerve cord (I), and the brain (J). In the CNS and brain, *UAS-6xEF* shows significantly larger cell volume than both control and *UAS-4xCC*. In the nerve cord, *UAS-6xEF* is only significantly larger than control. *UAS-4xCC* is not significantly larger than control in any measurement (*p ≤ 0.05, **p ≤ 0.01, ***p ≤ 0.001, Student two-tailed *t* test; *n* = 10 CNSs; ±SD). (K–L) NB5-6T lineages at larvae stage L1 in control and *lbe(K)-Gal4/UAS-6xEF* dissected CNSs. Boxed regions are magnified to the right. In control, no divisions are observed in NB5-6, neither in T nor A segments. In *6xEF* co-misexpression, dividing NBs and daughter cells can be observed in both T and A segments, and the lineage is larger. (M) Quantification of the number of cells in NB5-6T at stage L1 (*p ≤ 0.05, **p ≤ 0.01, ***p ≤ 0.001, Student two-tailed *t* test; *n* = 40 lineages; ±SD). (N) Cartoon depicting the NB5-6T lineage in control and in *lbe(K)-Gal4/UAS-6xEF*. The numerical data underlying this figure are included in S1 Data. Genotypes: (A, D, G–J) *insc-Gal4/+*. (B, E, G–J) *insc-Gal4/UAS-4xCC*. (C, F, G–J) *insc-Gal4/UAS-6xEF*. (K, M) *lbe(K)-Gal4*, *UAS-nls-myc-EGFP/+*. (L–M) *lbe(K)-Gal4*, *UAS-nls-myc-EGFP/UAS-6xEF*. A, abdominal; AFT, air-filled trachea; CNS, central nervous system; DAPI, 4′,6-diamidino-2-phenylindole; DC, daughter cell; Dpn, Deadpan; EF, Early Factor; *EGFP*, *Enhanced Green Fluorescent Protein*; *Gal4*, *Galactose4*; *insc*, *inscuteable*; *lbe(K)*, *ladybird early gene fragment K*; *myc*, *C-myc epitope tag*; NB, neuroblast; *nls*, *nuclear localization signal*; PH3, phosphorylated Ser10 on Histone-H3; Pros, Prospero; T, thoracic; *UAS*, upstream activating sequence.

In control animals, at late embryogenesis stage (air-filled trachea [AFT]), PH3 staining was typically observed in only a few lineages in the brain and occasional cells in the nerve cord (Fig 2A and 2G). In contrast, in *insc-Gal4/UAS-6xEF* animals, we observed a striking increase in proliferation, with extensive numbers of PH3 cells in both the brain and nerve cord (Fig 2C and 2G). Previous studies demonstrated that the EFs regulate a number of key cell-cycle factors [21, 23, 30]. Therefore, we wished to compare the *UAS-6xEF* effects to that of direct misexpression of key cell-cycle genes. To this end, we co-misexpressed the four cell-cycle genes *Cyclin E* (*CycE*), *Cyclin dependent kinase 2* (*Cdk2*), *E2F transcription factor 1* (*E2f1*), and *Dimerization partner* (*Dp*) (denoted *UAS-4xCC* herein) that previous studies have shown to be critical for *Drosophila* embryonic CNS proliferation [19, 35]. This co-misexpression (*insc-Gal4/UAS-4xCC*) also resulted in significantly increased proliferation, but strikingly, to a lesser degree than *UAS-6xEF* (Fig 2B and 2G). We furthermore noticed that the ectopic proliferation evident in the stage AFT nerve cord involved both dividing NBs and daughter cells (Fig 2A′–2C′).

To address the effects of misexpression on CNS size, we used 4′,6-diamidino-2-phenylindole (DAPI) nuclear staining to quantify total nuclear (cellular) volume. This revealed striking increase in CNS volume for *UAS-6xEF* and a trend upwards for *UAS-4xCC*, albeit not a significant one (Fig 2D–2F and 2H). Quantifying the nerve cord and brain separately, as anticipated for *UAS-6xEF* co-misexpression, we observed cellular volume expansion in the nerve cord (Fig 2I). Somewhat surprisingly, the expansion was even more pronounced for the brain (Fig 2J). *UAS-4xCC* showed an upwards trend in volume both in the nerve cord and the brain, but this was not significant (Fig 2I and 2J). Hence, both with respect to proliferation and CNS volume, *6xEF* co-misexpression is more potent than *4xCC* co-misexpression.

Focusing further on the *UAS-6xEF* co-misexpression, the presence of dividing NBs and daughter cells indicated that both the NB exit and the Type I->0 daughter cell proliferation switch was over-ridden by co-misexpression (Fig 2C′). To further address this notion, we turned to single-NB–lineage analysis, using the NB5-6 specific *lbe(K)-Gal4* driver [36]. NB5-6 NBs are generated by late St8 [12, 13] and commence proliferating in the Type I mode [3, 19, 36]; they then switch to Type 0 proliferation for several rounds until the NB exits the cell cycle and undergoes apoptosis (Fig 2N). In abdominal segments, Hox genes of the Bithorax Complex (BX-C) trigger an earlier Type I->0 switch and NB exit, resulting in smaller abdominal NB5-6 lineages (Fig 2K) [3, 12, 13, 34]. By St15, both thoracic and abdominal NB5-6 NBs have exited the cell cycle and undergone apoptosis. Hence, from St16 and onwards into AFT and L1 larval stages, there are no cell divisions observed in this lineage (Fig 2K, S4A Fig). In contrast, in *lbe(K)-Gal4/UAS-6xEF*, we observed ectopic NB and daughter cell divisions both at AFT and L1 (Fig 2L, S4B Fig). This resulted in a greatly expanded lineage size (Fig 2M, S4C Fig). These experiments demonstrate that EF co-misexpression can override both the Type I->0 switch and NB exit and drive aberrant continuing lineage progression (Fig 2N).

### EFs and Hox genes regulate each other

**Fig 3:**
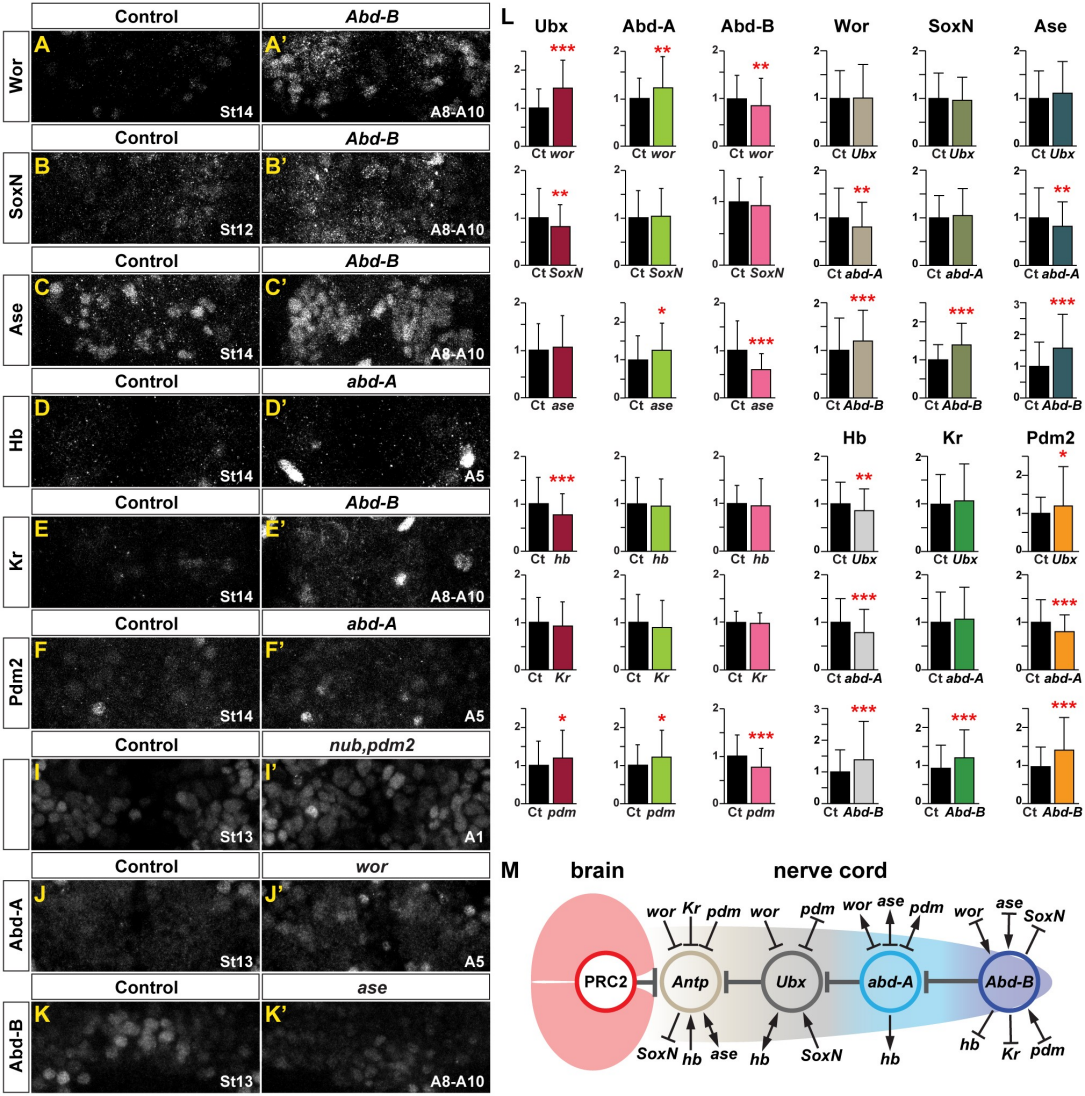
EFs and Hox genes regulate each other. (A–K′) Expression of Hox factors and EFs in control as well as in Hox and EF mutants. (L) Quantification of staining intensity of Hox factors and EFs in NBs in control and in EF and Hox mutants (see Materials and methods for details). St13 for Hox levels, St12 for SoxN levels, and St14 for the other five EF levels. Segments A1 for Ubx levels and *Ubx* mutants, A5 for Abd-A levels and *abd-A* mutants, and A8–A10 for Abd-B levels and *Abd-B* mutants (*p ≤ 0.05, **p ≤ 0.01, ***p ≤ 0.001, Student two-tailed *t* test; *n* = 3 CNSs; ±SD). (M) Cartoon summarizing the identified cross-regulation between Hox genes and EFs, based upon data herein and previous studies [21]. The numerical data underlying this figure are included in S1 Data. Genotypes: Control = *OregonR*. *Ubx* = *Ubx*^*1*^*/ Df(3R)Ubx109/Dp(3;3)P5*. *abd-A* = *abd-A*^*MX1*^. *Abd-B* = *Abd-B*^*D18*^. *ase* = *Df(1)ase-1*. *SoxN* = *SoxN*^*NC14*^*/Df(2L)Exel7040*. *wor* = *wor*^*4*^*/Df(2L)ED1054*. *hb* = *hb*^*P1*^, *hb*^*FB*^. *Kr* = *Kr*^*1*^, *Kr*^*CD*^. *nub*, *pdm2* = *Df(2L)ED773*. *abd-A*, *abdominal-A*; *Abd-B*, *Abdominal-B*; *Ase*, *Asense*; CNS, central nervous system; Ct, Control; Dpn, Deadpan; EF, Early Factor; Hb, Hunchback; Hox, Homeobox; Kr, Kruppel; NB, neuroblast; Pdm, POU domain; PRC2, Polycomb Repressor Complex 2; SoxN, SoxNeuro; St, Stage; *Ubx*, *Ultrabithorax*; Wor, Worniou.

Previous studies addressing the interplay between EF and Hox genes focused on the thorax and the *Antennapedia* (*Antp*) Hox gene [21]. This revealed that EFs and *Antp* regulate each other. Here, we addressed the interplay between EFs and the BX-C Hox genes *Ultrabithorax* (*Ubx*), *abdominal-A* (*abd-A*), and *Abdominal-B* (*Abd-B*) by analyzing EF and BX-C mutants for EF and BX-C expression. Expression in NBs was quantified in the pertinent segment for each mutant (Fig 3A–3L). Of the 36 analyses performed, no fewer than 22 showed significant effects on protein expression (Fig 3L). The most clear-cut interplay was between *Abd-B* and the EFs, in which *Abd-B* acted as a repressor of all six EFs. This interaction fits well with A8–A10 displaying the earliest Type I->0 daughter cell proliferation switch and earliest NB exit [7], with the strong antiproliferative role of *Abd-B* [3], and with A8–A10 showing the lowest levels of EFs along the A–P axis of the CNS (Fig 1D). In contrast, three of the EFs were activators of Abd-B. We also observed that *wor* and *nub*,*pdm2* repressed Ubx, and that *wor*, *ase*, and *nub*,*pdm2* repressed Abd-A. In contrast, *hb* and *SoxN* activated Ubx, and surprisingly, *abd-A* activated Wor, Ase, and Pdm2.

In summary, combined with the previous study of EF-*Antp* interactions [21], we find that out of the 48 possible interactions between EFs and *Antp*/BX-C, we find 29 interactions (Fig 3M). The majority of these (16) are repressive and fit with the general theme of EFs repressing Hox genes and vice versa. Surprisingly, 13 interactions involved EF activation of Hox factors and vice versa, perhaps pointing to negative feedback.

Previous studies, using Chromatin immunopurification-sequencing (ChIP-seq) and DNA adenine methyltransferase identification-sequencing (Dam-ID-seq), reveal that several of the EF and Hox factors bind to each other’s genes [22, 37–39] (www.modencode.org) (S1 Table). These findings suggest that the cross-regulation observed herein may result from direct transcriptional regulation.

### Elevated EF expression in the brain depends upon PRC2

**Fig 4:**
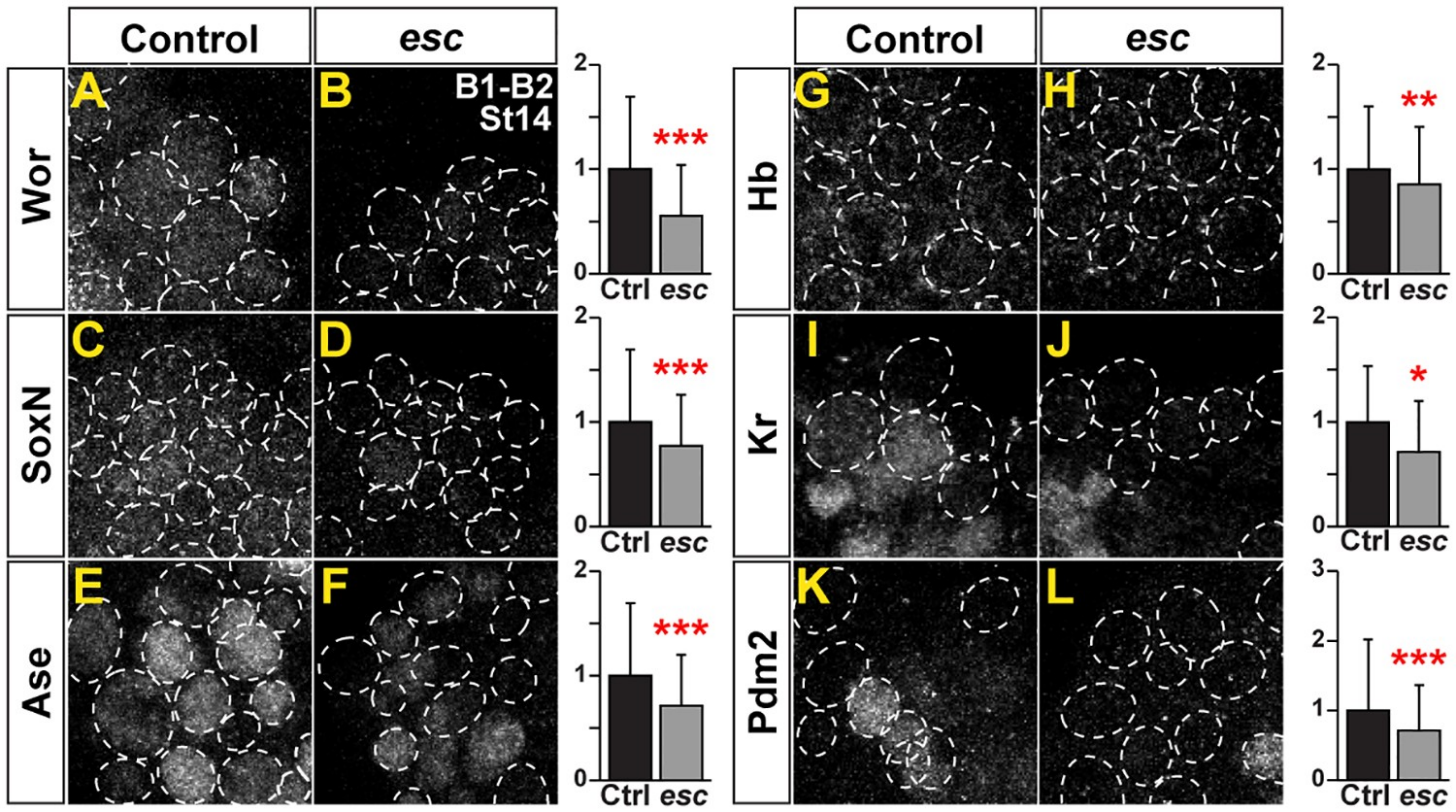
EF expression is reduced in PRC2 mutants. (A–L) Expression of EFs in B1–B2 NBs in control and *esc* maternal/zygotic mutants, St14. Dashed circles outline NBs, detected by Dpn. (Graphs to the right) Quantification of EF staining intensity in NBs, in control and *esc* maternal/zygotic mutants (*p ≤ 0.05, **p ≤ 0.01, ***p ≤ 0.001, Student two-tailed *t* test; *n* = 3 CNSs: *n* ≥ 300 NBs; ±SD). All six EFs are significantly down-regulated in *esc* mutants. The numerical data underlying this figure are included in S1 Data. Genotypes: (A, C, E, G, I, K) *OregonR*. (B, D, F, H, J, L) *esc*^*5*^ or *esc*^*21*^ over *esc*^*Df*^ (*Df(2L)Exel6030*). *esc*^*5*^/*esc*^*21*^ females crossed to *esc*^*Df*^ males. Ase, Asense; CNS, central nervous system; Ctrl, control; Dpn, Deadpan; EF, Early Factor; *esc*, *extra sex combs*; Hb, Hunchback; Kr, Kruppel; NB, neuroblast; Pdm, POU domain; PRC2, Polycomb Repressor Complex 2; SoxN, SoxNeuro; St, Stage; Wor, Worniou.

The *Drosophila* embryonic brain (B1–B2) does not express any of the Hox homeotic genes [7, 40]. A key regulatory system ensuring the repression of Hox genes in the brain is the PcG complex, and in particular the PRC2 [7, 41–44]. PRC2 is the key epigenetic complex responsible for adding the repressive H3K27me3 mark upon histone H3 [45, 46], and PRC2 is critical for the apparent lack of any Type I->0 daughter cell proliferation switches and the prolonged phase of NB proliferation observed in the brain [7]. To address the possible effect of PRC2 mutation on the elevated expression of EFs observed in the brain, we analyzed maternal and zygotic *extra sex combs* mutants (*esc*; mammalian *Embryonic Ectoderm Development* [EED]). Esc/EED is a key PRC2 component [45, 46], and *esc* maternal/zygotic mutants display nondetectable levels of H3K27me3 in the developing embryo accompanied by ectopic expression of Antp and BX-C in the entire brain (S5A–S5J Fig) [7]. As previously observed [7], in wild-type embryos, the brain showed stronger staining for the H3K27me3 mark (S5A Fig). In *esc* maternal/zygotic mutants, verified by nondetectable levels of H3K27me3 staining and ectopic Abd-B expression in the B1-B2 region (S5F and S5J Fig), we observed that all six EFs were significantly down-regulated in NBs (Fig 4A–4L).

### EF expression rescues PRC2 mutants

**Fig 5:**
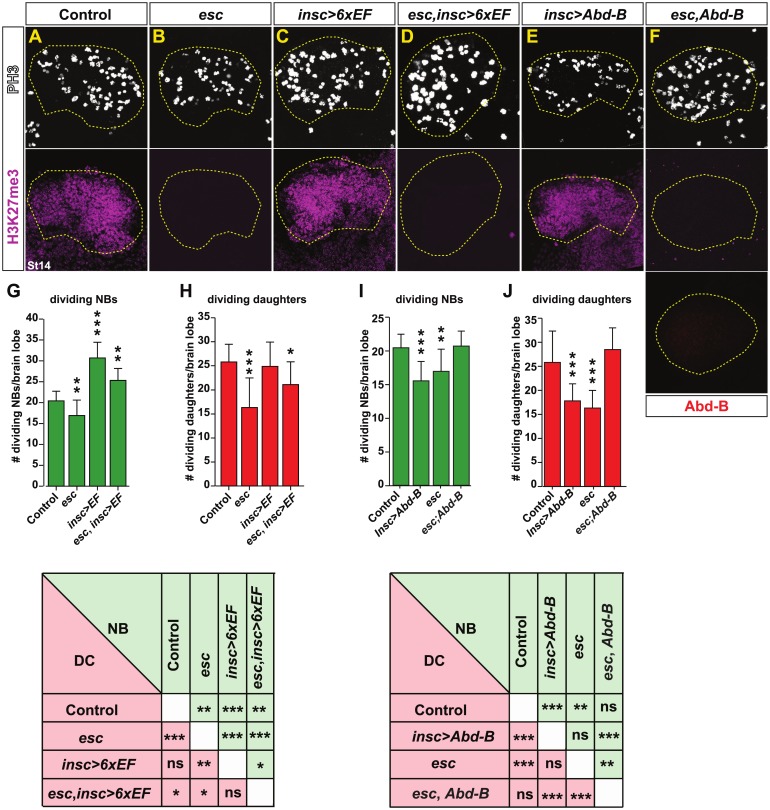
*6xEF* expression or *Abd-B* mutation rescues PRC2 mutants. (A–F) PH3 (proliferation) and H3K27me3 staining, as well as Abd-B (F) expression, in control, *esc*, and *esc* with *6xEF* cross-rescue, as well as *Abd-B* misexpression and *esc;Abd-B* double mutants, at St14 in segments B1–B3. (G–J) Quantification of dividing NBs and daughter cells, segments B1–B3, at St14 (*p ≤ 0.05, **p ≤ 0.01, ***p ≤ 0.001, Student two-tailed *t* test; *n* = 10 embryos; ±SD). The numerical data underlying this figure are included in S1 Data. Genotypes: (A) *OregonR*. (B) *esc*^*5*^ or *esc*^*21*^ over *esc*^*Df*^ (*Df(2L)Exel6030*). (C) *insc-Gal4/UAS-6xEF*. (D) *esc*^*5*^ or *esc*^*21*^ over *esc*^*Df*^ (*Df(2L)Exel6030*), *insc-Gal4/UAS-6xEF*. (E) *insc-Gal4/UAS-Abd-B*. (F) *esc*^*5*^ or *esc*^*21*^ over *esc*^*Df*^ (*Df(2L)Exel6030*); *Abd-B*. *Abd-B*, *Abdominal-B*; EF, Early Factor; *esc*, *extra sex combs*; *Gal4*, *Galactose4*; DC, daughter cell; H3K27me3, Histone 3 K27 trimethylation; *insc*, *inscuteable*; NB, neuroblast; ns, not significant; PH3, phosphorylated Ser10 on Histone-H3; PRC2, Polycomb Repressor Complex 2; St, Stage; UAS, upstream activating sequence.

Having found that EFs are down-regulated in *esc* mutants, we wanted to test whether EFs transgenic expression could cross-rescue *esc* mutants. To this end, we co-misexpressed all six EFs, driven from the *insc-Gal4* driver, in an *esc* maternal/zygotic mutant background. Strikingly, we found that transgenic *6xEF* coexpression could completely override the reduced NB and daughter cell proliferation observed in *esc* mutants (Fig 5A–5C, 5G and 5H). Hence, the strong antiproliferative effect of PRC2 upon brain development could be over-ridden by *6xEF* transgenic coexpression.

### Mutation of *Abd-B* rescues PRC2 mutants

The Type I->0 daughter cell proliferation switch and NB exit both occur first in the A8–A10 region, and *Abd-B* plays a prominent role in triggering these events [3]. The repressive role of *Abd-B* on all six EFs (Fig 3M) and the extension of Abd-B expression into B1-B2 in *esc* mutants (S5E and S5J Fig) [7], prompted us to address the extent to which reduced proliferation in *esc* mutants result from ectopic brain expression of *Abd-B*.

First, we addressed the potency of *Abd-B* in triggering the Type I->0 switch and the NB exit in the wild-type background by misexpressing it in the developing brain using *insc-Gal4*. We observed striking reduction of both NB and daughter cell proliferation in the B1–B3 segments (Fig 5E, 5I and 5J). This demonstrates that *Abd-B* is sufficient, at least in part, to impose a posterior nerve cord proliferation behavior on the brain. Next, we addressed the role of *Abd-B* in an *esc* mutant background by generating maternal and zygotic homozygous *esc* mutants simultaneously zygotically homozygous for *Abd-B* (*esc; Abd-B*). Strikingly, we found that the reduction of NB and daughter cell proliferation observed in *esc* mutants was rescued by *Abd-B* homozygosity (Fig 5F, 5I and 5J). In fact, surprisingly, *Abd-B* homozygosity restored NB and daughter cell proliferation back to wild-type levels (Fig 5I and 5J).

## Discussion

### Gradient of EF expression promotes anterior CNS expansion

**Fig 6:**
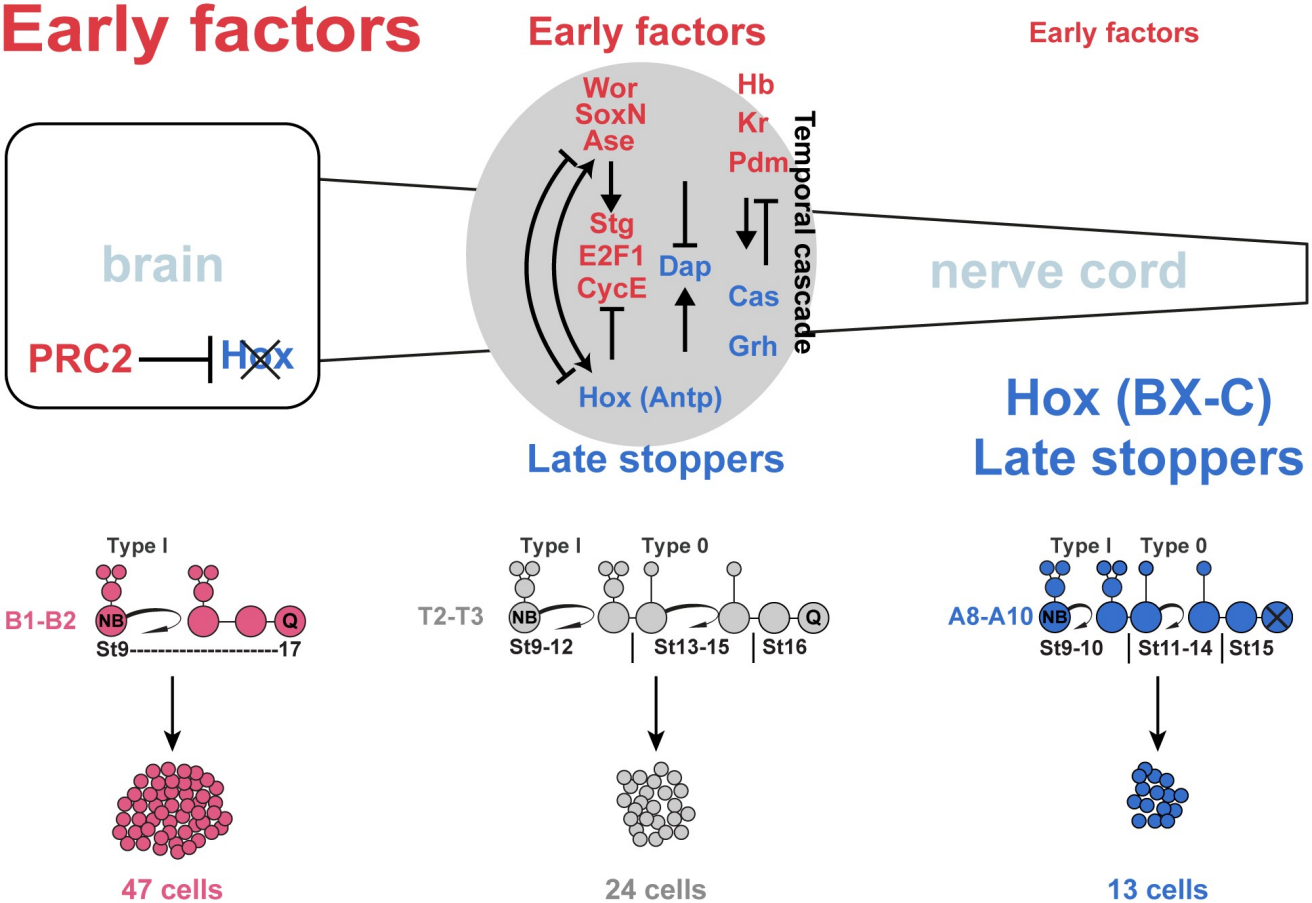
Anterior enhancement of neural stem cell program by PRC2–Hox. EFs generate NBs and drive NB and daughter proliferation. In the nerve cord, EF activity is counteracted by Hox homeotic genes, forming a gradient of EF expression, NB proliferation, and the Type I->0 daughter proliferation switch. This results in a gradient of lineage size along the nerve cord. In the brain, PRC2 prevents Hox expression, allowing for further elevation of EF expression and thereby extended NB proliferation and an absence of the Type I->0 daughter proliferation switch. This results in even larger lineages in the brain (see text for details). Antp, Antennapedia; Ase, Asense; BX-C, Bithorax Complex; Cas, Castor; CycE, Cyclin E; Dap, Dacapo; EF, Early Factor; Grh, Grainy head; Hb, Hunchback; Hox, Homeobox; Kr, Kruppel; NB, neuroblast; Pdm, POU domain; PRC2, Polycomb Repressor Complex 2; SoxN, SoxNeuro; St, Stage; Stg, String; Wor, Worniou.

The nerve cord, specifically T1–A10, shows a gradient of NB proliferation and Type I->0 daughter cell proliferation switches [3, 4]. This is controlled by the graded expression of Hox genes and the increasingly antiproliferative role of posteriorly expressed Hox genes [3, 7]. In contrast, the brain is “hyperproliferative,” with prolonged NB proliferation and an apparent absence of the Type I->0 switch [7]. Here, we find significant differences when comparing EF levels along the A–P axis, with elevated and extended expression in the brain and a gradient along the nerve cord. Together with the mutant and misexpression data (herein; [21]), this supports a model in which graded EF levels are key for generating graded proliferation along the CNS A–P axis and, at elevated levels, drives brain-type proliferation (Fig 6).

The Type I->0 daughter cell proliferation switch and the precise NB cell-cycle exit depend upon balanced levels of four key cell-cycle genes: *Cyclin E* (*CycE*), *string* (*stg*; mammalian *Cdc25a/b/c*), *E2f1*, and *dacapo* (*dap*; *Cdkn1a/b/c*) [19, 35]. Previous studies of EFs, Hox genes, and PRC2 demonstrated regulatory links to these cell-cycle genes [3, 7, 19, 21, 23, 30]. This regulation results in both temporal and spatial changes in cell-cycle gene expression. Hence, cell-cycle “drivers” (CycE, E2f1, Stg) are elevated in early NBs when compared to late and elevated in brain when compared to nerve cord, while “stoppers” (Dap) show the inverse profile [7, 19]. Surprisingly, the co-misexpression of *CycE*, *Cdk2*, *Dp*, and *E2f1* (*UAS-4xCC*) gave considerably weaker effects than that of *6xEF* co-misexpression. One explanation for this weaker effect may be that EFs are involved in also regulating *stg* (“driver”) and *dap* (“stopper”) [21, 23, 30], which were not simultaneously misexpressed/mutated in these experiments.

In addition to Type I and Type 0, a third type of embryonic NB behavior was recently identified: Type II NBs [1, 6]. These NBs generate daughter cells that divide multiple times. Of the 1,200 NBs in the embryo, and 228 in the brain (B1–B3), only 16—eight in each B1 segment—have been identified. Strikingly, recent analysis reveal that Type II NBs do not express several of the EFs studied herein, e.g., Ase, Hb, and Kr, while they do express Pdm, Cas, Grh, and Pointed-P1 [1, 6]. This suggests that while Type I NB identity (including switching to Type 0 in the nerve cord) is specified by a common EF program, the Type II NBs are specified by a partly nonoverlapping genetic program.

### A PcG->Hox->EF program promoting brain expansion

The graded proliferation observed along the A–P axis of the CNS is controlled by graded Hox input in the nerve cord and an absence of Hox expression in the brain (B1–B2) [3, 4, 7, 19, 34]. The graded proliferation is mediated by graded EF expression, also under the influence of graded Hox gene expression. All three components—i.e., graded proliferation, Hox expression, and EF expression—are controlled by PRC2 (this work; [3, 7, 21]). To begin addressing this interplay, we conducted two types of cross-rescue experiments. First, we found that we could rescue *esc* by transgenic re-elevation of *6xEF*s, demonstrating that in spite of nondetectable levels of the H3K27me3 mark and aberrant Hox expression in the brain, EFs can still restore brain-type proliferation. Second, we found that we could rescue the reduced brain proliferation in *esc* mutants by merely removing one Hox gene: *Abd-B*. While this second finding may at first glance be surprising, given the many possible roles of PRC2 and the ectopic expression of Hox genes in *esc* mutant brains, the rescue fits with several observations. First, of the four nerve cord Hox genes, *Abd-B* has the most prominent effect on proliferation in the nerve cord, explaining why A8–A10 display the earliest Type I->0 switch and NB exit, and hence the smallest lineages [3]. Second, *Abd-B* has strongly repressing effects on all six EFs, and, logically, EF expression is at its lowest in A8–A10. Third, in *esc* mutants, Abd-B is robustly expressed throughout the brain. Fourth, *Abd-B* misexpression in the brain results in reduced proliferation. Fifth, in the mouse, the posteriormost Hox gene in the B cluster, *Hoxb13* (an *Abd-B* orthologue), has strong effects on spinal cord proliferation [47]. Moreover, misexpression of *Hoxb13* in the chick telencephalon also suppressed proliferation and showed stronger effects than the more anteriorly expressed *Hoxb9* gene [7]. In combination, the potency of *Abd-B* and perhaps the *Abd-B* paralog group in general (the most posteriorly expressed Hox group) combine to explain why *esc* could be rescued by removal of only one Hox gene. In combination, the regulatory interplay between Hox genes and EFs, the EF cross-rescue of *esc*, the *Abd-B* rescue of *esc*, and the PRC2 regulation of EF combine to support the idea that the main role of PRC2, with regards to promoting brain proliferation, is to repress Hox gene expression in the brain, thereby allowing for elevated EF expression.

In humans, three of the four main components of PRC2, including *EED* (*Drosophila esc*), *EZH2*, and *SUZ12*, are predicted to be haploinsufficient [48]. In line with this notion, several related human developmental syndromes, including the Weaver, Weaver-like, Cohen-Gibson, and Overgrowth and Intellectual Disability syndromes, have been linked to heterozygous, usually de novo, mutations in *EED*, *EZH2*, or *SUZ12* [49–60]. In some cases, these mutations were shown to result in reduced H3K27me3 activity [51, 55]. These syndromes manifest with a range of peripheral overgrowth phenotypes but also neurological defects, involving delayed speech and psychomotor development as well as intellectual disability. MRI analysis has revealed a range of brain morphology anomalies, including polymicrogyria and white matter volume loss [51, 52]. It is tempting to speculate that our surprising finding that mutation in one single Hox gene could rescue *esc* mutants may point to new avenues of investigation for these syndromes.

### Is the PcG->Hox->EF program conserved during evolution?

A defining feature of bilaterians is the condensation of neural cells into a centralized and contiguous tissue: the CNS. In most, if not all, bilaterians a common property of the CNS is that the anterior part, the brain, is larger than the nerve cord. The brain and the nerve cord express distinct sets of highly conserved patterning genes, with the brain expressing a number of “brain-specific” genes and the nerve cord expressing Hox homeotic genes [44, 61–63]. The distinct patterning gene expression, as well as other aspects of nervous system development and evolution, has recently prompted the idea of a separate evolutionary origin of these CNS regions [61, 63–65]. Intriguingly, in arthropods such as *Drosophila*, the initial development of the brain and nerve cord is separate, only to merge during subsequent embryogenesis [66]. The finding of distinct proliferation patterns in the *Drosophila* brain versus nerve cord, with the brain using Type I and Type II daughter proliferation and extended NB proliferation [1, 6, 7] and the nerve cord displaying the Type I->0 daughter proliferation switch and earlier NB exit [3, 19], lends further support for the notion of separate evolutionary origins for the brain and nerve cord.

In parallel to A–P patterning of the developing CNS, neural stem cells are generated along the entire neuraxis. This study, and others, point to the fact that in *Drosophila*, there is a common program for establishing and driving NBs along the entire neuraxis. Studies in mammals have revealed that a number of the genes involved in the *Drosophila* neural stem cell program are evolutionarily conserved. Specifically, SoxN and D (SoxB family members) are orthologues of mammalian Sox1, -2, and -3, which are critical for CNS development [67]. The *wor*/*sna*/*esg* orthologue *Snail1* is important for proliferation of mouse neural stem cells [68] and important for neural stem cell reprogramming [69]. The *ase* orthologue *Achaete-scute complex homolog 2* (*Ascl2*) is critical for adult neurogenesis and proliferation [70, 71]. Herein, we demonstrate that the EFs, critical activators of the *Drosophila* neural stem cell program, are expressed in an A–P gradient along the developing CNS and that this gradient plays a key role in driving the wedge-like structure of the CNS, with its prominent anterior expansion. What is the evidence for a similar A–P gradient of the mammalian orthologues of the *Drosophila* neural stem cell program? To our knowledge, the A–P aspect of the mammalian neural stem cell program has not been extensively addressed. However, recent transcriptome analysis of the developing mouse forebrain versus spinal cord demonstrated elevated anterior levels of Sox1, -2, and -3 in the forebrain [7]. It is tempting to speculate that the conserved role of the PcG->Hox program in controlling proliferation [7] also extends into the conserved regulation of graded levels of an evolutionarily conserved neural stem cell (EF) program, acting to drive the expansion of the anterior CNS, common to most, if not all bilaterians.

## Materials and methods

### Fly stocks

#### Mutants and reporters

*wor*^*4*^ (Bloomington Drosophila Stock Center BL #25170). *wor*^*Df*^ = *Df(2L)ED1054* (BL #24112). *SoxN*^*NC14*^ (BL #9938). *SoxN*^*Df*^ = *Df(2L)Exel7040* (BL #7811). *hb*^*P1*^, *hb*^*FB*^ and *Kr*^*1*^, *Kr*^*CD*^ [72] (provided by Chris Q. Doe, University of Oregon, Eugene, OR, USA). *ase*^*Df*^ = *Df(1)ase-1* (BL #104). *Df(2L)ED773* (removes both *nub* and *pdm2*; BL #7416). *Df(3R)Ubx109/Dp(3;3)P5* (BL #3486). *abd-A*^*MX1*^ (BL #3057). *Ubx*^*1*^ (BL #2866). *Abd-B*^*D18*^, mutant for both *m* and *r* isoforms [73] (obtained from Ernesto Sanchez-Herrero, CBM, Madrid, Spain). *lbe(K)-EGFP* [74]. *UAS-nls-myc-EGFP* (referred to as *UAS-nmEGFP*) [75]. *esc*^*5*^ (BL #3142). *esc*^*21*^ (BL #3623). *esc*^*Df*^ = *Df(2L)Exel6030* (BL #7513). *UAS-myr-mRFP* (mobilized version of BL #7119 on the third chr) [35].

#### *UAS* transgenes

*UAS-E2f*, *UAS-Dp* (BL #4770, BL #4774). *UAS-CycE*, *UAS-cdk2* (previously available from Bloomington). *UAS-6xEF* = *UAS-hb-myc* (*22A*), *UAS-ase-myc (28E*), *UAS-Kr-V5* (*53B*); *UAS-wor-FLAG* (*65B*), *UAS-nub-HA* (*68A*), *UAS-SoxN-V5* (*89E*) [21]. *UAS-Abd-B* [3].

#### *Gal4* drivers

*insc*^*Mz1407*^ (referred to as *insc-Gal4*; BL #8751). *lbe(K)-Gal4* [36].

Mutants were maintained over *GFP*- or *YFP*-marked balancer chromosomes. Staging of embryos was performed according to Campos-Ortega and Hartenstein [76].

### Generation of *esc* maternal/zygotic mutants

From a cross between esc^5^ and *esc*^*21*^ mutants, *esc*^*5*^*/esc*^*21*^ females were selected. These females were crossed to *esc*^*Df*^ males (*Df(2L)Exel6030*) and set for embryo collection. *esc* homozygous mutant embryos, i.e., *esc*^*5*^*/escDf* or *esc*^*21*^*/esc*^*Df*^ maternal-zygotic mutant embryos, were identified by lack of the YFP-marked balancer chromosome.

### Immunohistochemistry

Immunohistochemistry was conducted as previously described [21]. Primary antibodies were guinea pig anti-Dpn (1:1,000) and rat anti-Dpn (1:500) [74]; rabbit anti-phospho H3-Ser10 (PH3) (1:1,000) (cat. no. ab5176, Abcam, Cambridge, UK); rat anti-PH3-ser28 (1:1,000; cat. no. ab10543, Abcam, Cambridge, UK); chicken anti-GFP (1:2,000; cat. no. ab13970, Abcam); rat mAb anti-GsbN (1:10) [77] (provided by Robert Holmgren, Northwestern University, Evanston, IL, USA); rabbit anti-Abd-A (1:100) (provided by Maria Capovila, CNRS, Sophia Antipolis, France); mouse mAb anti-Ubx (1:10), mAb anti-Abd-A (1:10), mAb anti-Abd-B (1:10), mAb anti-Pros MR1A (1:10) (Developmental Studies Hybridoma Bank, Iowa City, IA, US); rabbit anti-Ase (1:1,000; provided by Yuh-Nung Jan, UCSF, San Francisco, USA); rabbit anti-SoxN (provided by Steve Russell, Cambridge University, Cambridge, UK); rat anti-Wor (1:1) (provided by Chris Q. Doe, University of Oregon, Eugene, OR, USA); rabbit anti-Hb and anti-Kr (1:500) (provided by Ralf Pflanz, MPI, Göttingen, Germany); mouse anti-Nub (1:100) (also detects Pdm2; provided by Steve Cohen and Hector Herranz, Temasek Life Sci, Singapore, Singapore); rat anti-Pdm2 (1:500; cat. no. ab201325, Abcam); rabbit anti-H3K27me3 (1:500; cat. no. 9733, Cell Signaling Technology, Danvers, MA, USA).

### Confocal imaging

For fluorescent images, we used Zeiss LSM700 or Zeiss LSM800 confocal microscopes (Zeiss, Oberkochen, Germany) and merged confocal stacks using LSM software or Fiji software [78]. Images and graphs were compiled using Adobe Illustrator.

### Quantification of CNS volume

Embryos were fixed for 20 minutes in 4% PFA. After fixation, immunostaining was performed as previously described [21]. Embryos were stained with DAPI (cat. no. D9564, Sigma-Aldrich, Sweden AB, Stockholm, Sweden) at 1 μg/ml together with the secondary antibody incubation. An ImageJ language-based semiautomated macro was written and used to quantify the CNS volume using the Fiji software [78].

### Quantification of proliferation

Embryos were dissected at precise stages, and mitotic NBs and daughter cells—as identified by PH3, Dpn, and Pros—were counted within each segment. An ImageJ language-based semiautomated macro was written and used for quantification with Fiji software [78].

### Protein intensity measurements

Fluorescent images were analyzed using Zeiss LSM800 confocal microscopes, and Fiji software was used to visualize confocal stacks. To ensure identical staining conditions, control and mutant/misexpression embryos were dissected on the same slide and scanned using the same confocal settings. Dpn was used to identify the NBs. The integrated density (mean pixel intensity × area occupied by the signal) of individual cells was measured using Fiji software, focusing on a single 1-μm–thick confocal layer encompassing the center of the cell. The mean in control was set to 1, and experimental data were normalized to control.

### Statistical analysis

Two-tailed Student *t* test was performed using Microsoft Excel 2016 or IBM SPSS V25.0 software (for specific statistical test used, see text and figures). Significance of *p* ≤ 0.05 is indicated with one star (*), *p* ≤ 0.01 with two stars (**), *p* ≤ 0.001 with three stars (***), and nonsignificant with “ns.” Microsoft Excel 2010 was used for data compilation and graphical representation. Figures and graphs were compiled using Adobe Photoshop and Adobe Illustrator.

## Supporting information

S1 Fig Expression of EFs is higher in the brain. (A–C) Mean intensity of EF protein levels along A–P axis, adjusted to their expression levels in T2–T3 (*p ≤ 0.05, **p ≤ 0.01, ***p ≤ 0.001, Student two-tailed *t* test; *n* = 4 embryos; 32 segments; ±SD). (D) Mean intensity of EF protein levels in B2–B3, adjusted to their expression levels in T2–T3 at St11 (*p ≤ 0.05, **p ≤ 0.01, ***p ≤ 0.001, Student two-tailed *t* test; *n* = 3 embryos; 6 segments; ±SD). For each factor, dissected embryos were stained on the same slide, and all CNS regions were scanned in each embryo. The numerical data underlying this figure are included in S1 Data. Genotypes: (A–D) *OregonR*. A–P, anterior–posterior; CNS, central nervous system; EF, Early Factor.
(PDF)

S2 Fig EFs are necessary for NB and daughter cell proliferation. (A) Staining for Pros, Dpn, and PH3 allows for the identification of dividing NBs (asymmetric Pros, Dpn+, PH3+) and dividing daughter cells (cytoplasmic Pros, Dpn negative, PH3+). (B–C) Proliferation in control and *ase*, at St14 in segment A1, reveals an apparent reduction in dividing cells in *ase*. (D–E) Quantification of dividing NBs and daughter cells in control and EF mutants, segments B1–B2, A1, A5, and A8–A10, at St14. With a few exceptions, proliferation of both NBs and daughter cells is reduced in EF mutants in both the brain and abdomen (see text for details) (*p ≤ 0.05, **p ≤ 0.01, ***p ≤ 0.001, Student two-tailed *t* test; *n* = 10 embryos; 60 segments; ±SD). The numerical data underlying this figure are included in S1 Data. Genotypes: (A–B) *OregonR*. (C) *ase* = *Df(1)ase-1*. (D–E) ase = *Df(1)ase-1*. *SoxN* = *SoxN*^*NC14*^/*Df(2L)Exel7040*. *wor* = *wor*^*4*^/*Df(2L)ED1054*. *hb* = *hb*^*P1*^, *hb*^*FB*^. *Kr* = *Kr*^*1*^, *Kr*^*CD*^. *nub*, *pdm2* = *Df(2L)ED773*. Ase, Asense; Dpn, Deadpan; EF, Early Factor; Hb, Hunchback; Kr, Kruppel; NB, neuroblast; Nub, Nubbin; Pdm, POU domain; PH3, phosphorylated Ser10 on Histone-H3; Pros, Prospero; St, Stage; SoxN, SoxNeuro; Wor, Worniou.
(PDF)

S3 Fig Combinatorial misexpression of the six EFs. (A–N) Embryonic fillets, showing expression of RFP and the six EFs, in control and *insc-Gal4/UAS* embryos, at St15. (H) RFP expression shows that *insc-Gal4* drives expression in the entire CNS. (B–G, I–N) *insc-Gal4/UAS-6xEF* embryos reveal elevated expression of all six EFs in the CNS. (O–Z) Expression of the six EFs in control and *insc-Gal4/UAS-6xEF* embryos, thoracic segment T2, at St15, NB layer (identified by Dpn staining). *insc-Gal4* drives elevated EF expression in NBs. Genotypes: (A–G, O–Q, U–W) *OregonR*. (H–N, R–T, X–Z) *insc-Gal4/UAS-6xEF*. CNS, central nervous system; EF, Early Factor; *insc-Gal4*, XXX; NB, neuroblast; RFP, Red Fluorescent Protein; St, Stage; *UAS*, upstream activating sequence.
(PDF)

S4 Fig EF co-misexpression overrides the Type I->0 switch and NB exit. (A–B) NB5-6T lineages at stage AFT in control and *lbe(K)-Gal4/UAS-6xEF* dissected CNSs. Boxed regions are magnified to the right. In control, no divisions are observed in NB5-6, neither in T nor A segments. In *6xEF* co-misexpression, dividing NBs and daughter cells can be observed in both T and A segments, and the lineage is larger. (C) Quantification of the number of cells in NB5-6T at stage AFT (*p ≤ 0.05, **p ≤ 0.01, ***p ≤ 0.001, Student two-tailed *t* test; *n* = 40 lineages; ±SD). The numerical data underlying this figure are included in S1 Data. Genotypes: (A) *lbe(K)-Gal4*, *UAS-nls-myc-EGFP*/+. (B) *lbe(K)-Gal4*, *UAS-nls-myc-EGFP/UAS-6xEF*. A, abdominal; AFT, air-filled trachea; EF, Early Factor; *EGFP*, Enhanced Green Fluorescent Protein; *Gal4*, *Galactose4*; *lbe(K)*, *ladybird early gene fragment K*; *myc*, *C-myc epitope tag*; NB, neuroblast; *nls*, *nuclear localization signal*; T, thoracic; *UAS*, upstream activating sequence.
(PDF)

S5 Fig PRC2 mutants display nondetectable levels of H3K27me3 and anterior Hox expression. (A–J) Expression of H3K27me3, Antp, Ubx, Abd-A, and Abd-B in control and *esc* maternal/zygotic mutants, St15. Dashed lines outline the CNS. In *esc* mutants, there are nondetectable levels of H3K27me3, and all four Hox factors are expressed along the entire A–P axis, including in the brain. The numerical data underlying this figure are included in S1 Data. Genotypes: (A–E) *OregonR*. (F-J) *esc*^*5*^ or *esc*^*21*^ over *esc*^*Df*^ (*Df(2L)Exel6030*). *abd-A*, *abdominal-A*; *Abd-B*, *Abdominal-B*; *Antp*, *Antennapedia*; A–P, anterior–posterior; CNS, central nervous system; *esc*, *extra sex combs*; Hox, Homeobox; H3K27me3, Histone 3 K27 trimethylation; PRC2, Polycomb Repressor Complex 2; St, Stage; *Ubx*, *Ultrabithorax*.
(PDF)

S1 Table Summary of previously published genome-wide DNA binding. Previous studies, using ChIP-seq and Dam-ID-seq, have revealed binding of EFs to Hox genes and of Ubx to EF genes [22, 37–39] (www.modencode.org). ChIP-seq, Chromatin immunoprecipitation-sequencing; Dam-ID-seq, DNA adenine methyltransferase identification-sequencing; EF, Early Factor; Hox, Homeobox; *Ubx*, *Ultrabithorax*.
(XLSX)

S1 Data Numerical data underlying figures. (XLSX)

## Abbreviations

A: abdominal
*abd-A*: *abdominal-A*
*Abd-B*: *Abdominal-B*
AFT: air-filled trachea
*Antp*: *Antennapedia*
*Ascl2*: *Achaete-scute complex homolog 2*
Ase: Asense
A–P: anterior–posterior
bHLH: basic helix-loop-helix
BX-C: Bithorax complex
Cas: Castor
Cdk2: Cyclin dependent kinase 2
ChIP-seq: Chromatin immunoprecipitation-sequencing
CNS: central nervous system
*CycE*: *Cyclin E*
D: Dichaete
Dam-ID-seq: DNA adenine methyltransferase identification-sequencing
Dap: Dacapo
DAPI: 4′,6-diamidino-2-phenylindole
DC: daughter cell
Dp: Dimerization partner
Dpn: Deadpan
EED: Embryonic Ectoderm Development
EF: Early Factor
*EGFP*: *Enhanced Green Fluorescent Protein*
*esc*: *extra sex combs*
E2f1: E2F transcription factor 1
*Gal4*: *Galactose4*
Grh: Grainy head
Hb: Hunchback
Hox: Homeobox
H3K27me3: Histone 3 K27 trimethylation
*insc*: *inscuteable*
Kr: Kruppel
*lbe(K)*: *ladybird early gene fragment K*
L1: larval stage 1
*myc*: *C-Myc epitope tag*
NB: neuroblast
*nls*: *nuclear localization signal*
Nub: Nubbin
PcG: Polycomb Group
Pdm: POU domain
PH3: phosphorylated Ser10 on Histone-H3
PRC2: Polycomb Repressor Complex 2
Pros: Prospero
SoxB: sex determining region Y-box B
SoxN: SoxNeuro
St: Stage
Stg: String
T: thoracic
UAS: upstream activating sequence
*Ubx*: *Ultrabithorax*
Wor: Worniou
