# Supplementary material for: Brain expansion promoted by polycomb-mediated anterior enhancement of a neural stem cell proliferation program
Source: PLoS Biol. 2019 Feb 26;17(2):e3000163. doi: 10.1371/journal.pbio.3000163 (PMC6407790; doi:10.1371/journal.pbio.3000163)

Supplemental Figure 1

Expression of Early Factors is higher in the brain

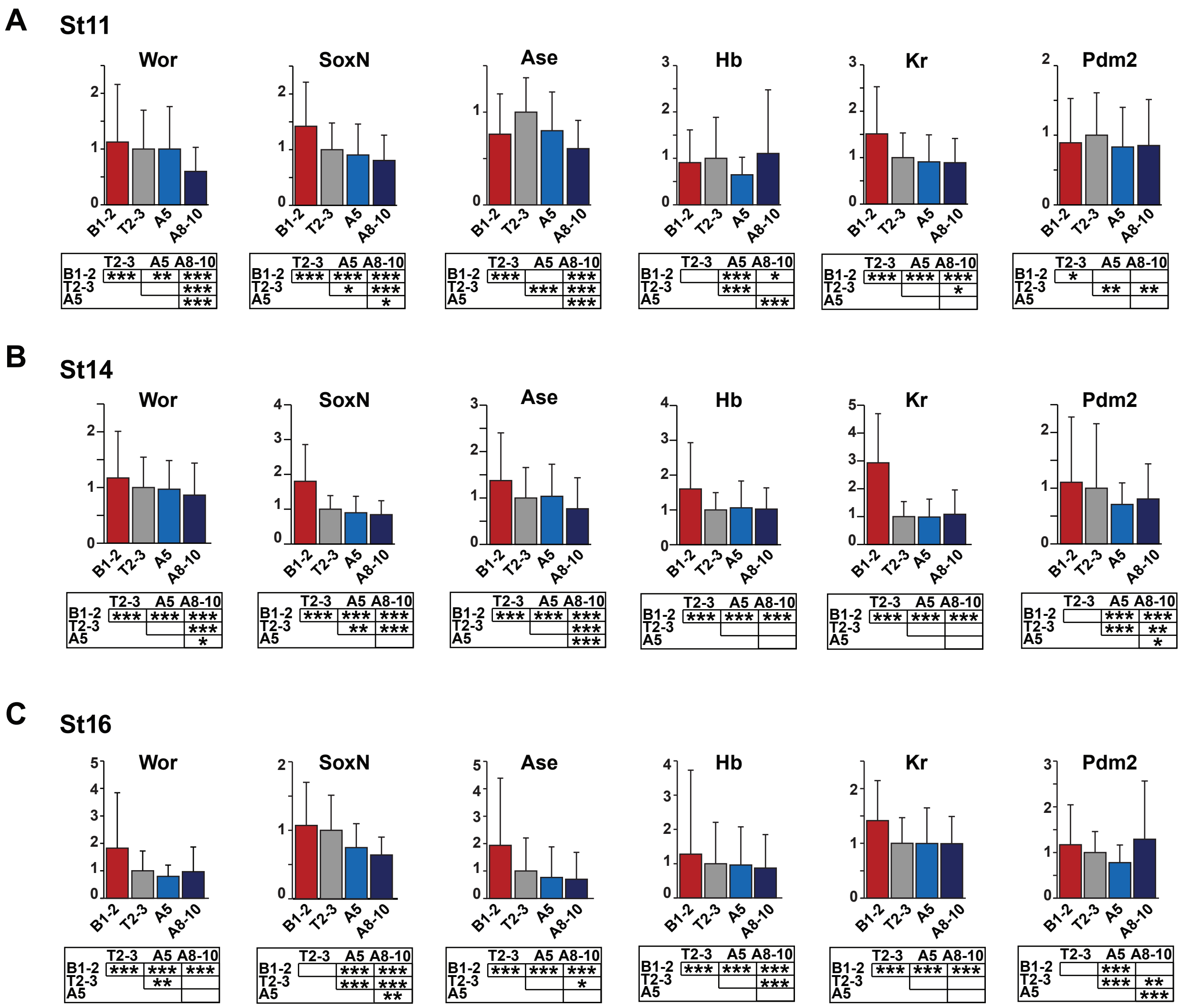

D

EF expression levels in B1-B2 go down over time

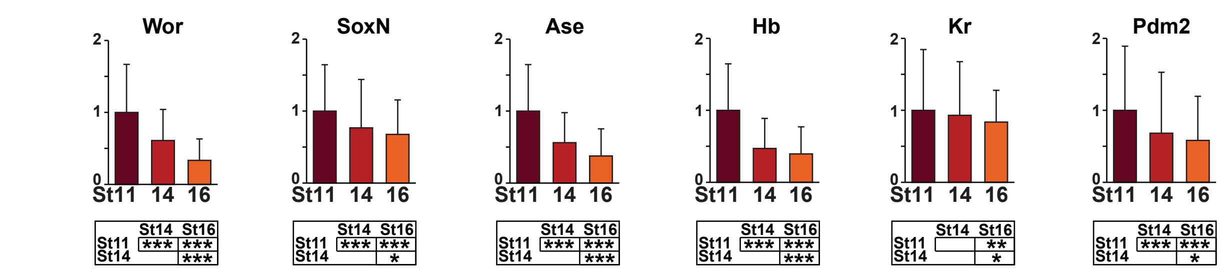

Supplement: S1 Fig — (A–C) Mean intensity of EF protein levels along A–P axis, adjusted to their expression levels in T2–T3 (*p ≤ 0.05, **p ≤ 0.01, ***p ≤ 0.001, Student two-tailed t test; n = 4 embryos; 32 segments; ±SD). (D) Mean intensity of EF protein levels in B2–B3, adjusted to their expression levels in T2–T3 at St11 (*p ≤ 0.05, **p ≤ 0.01, ***p ≤ 0.001, Student two-tailed t test; n = 3 embryos; 6 segments; ±SD). For each factor, dissected embryos were stained on the same slide, and all CNS regions were scanned in each embryo. The numerical data underlying this figure are included in S1 Data. Genotypes: (A–D) OregonR. A–P, anterior–posterior; CNS, central nervous system; EF, Early Factor. (PDF) [file pbio.3000163.s001.pdf]
