# Supplementary figures and images for: Brain expansion promoted by polycomb-mediated anterior enhancement of a neural stem cell proliferation program
Source: PLoS Biol. 2019 Feb 26;17(2):e3000163. doi: 10.1371/journal.pbio.3000163 (PMC6407790; doi:10.1371/journal.pbio.3000163)

# Supplemental Figure 2

## Early Factor mutants show reduced CNS proliferation

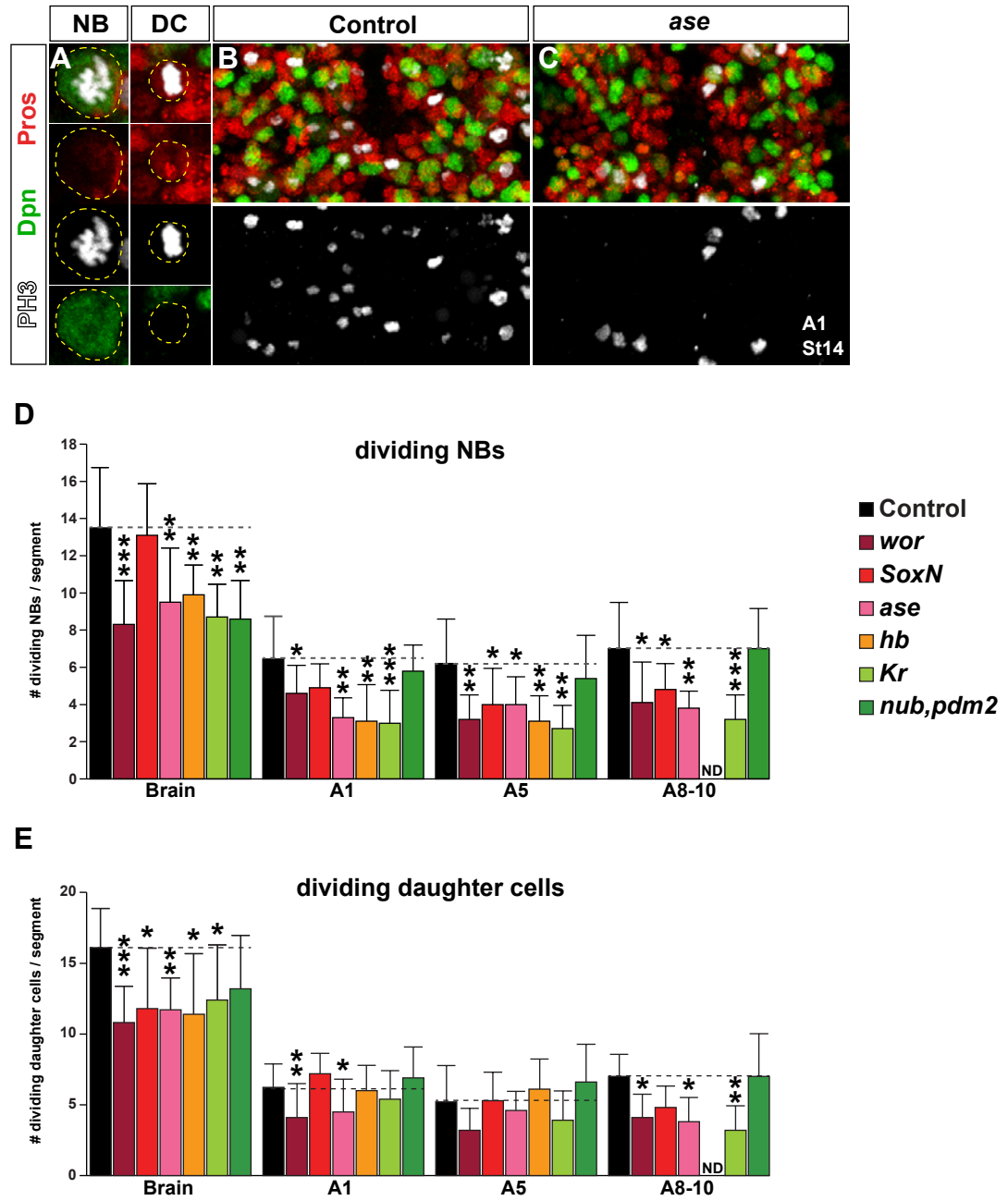

Supplement: S2 Fig — (A) Staining for Pros, Dpn, and PH3 allows for the identification of dividing NBs (asymmetric Pros, Dpn+, PH3+) and dividing daughter cells (cytoplasmic Pros, Dpn negative, PH3+). (B–C) Proliferation in control and ase, at St14 in segment A1, reveals an apparent reduction in dividing cells in ase. (D–E) Quantification of dividing NBs and daughter cells in control and EF mutants, segments B1–B2, A1, A5, and A8–A10, at St14. With a few exceptions, proliferation of both NBs and daughter cells is reduced in EF mutants in both the brain and abdomen (see text for details) (*p ≤ 0.05, **p ≤ 0.01, ***p ≤ 0.001, Student two-tailed t test; n = 10 embryos; 60 segments; ±SD). The numerical data underlying this figure are included in S1 Data. Genotypes: (A–B) OregonR. (C) ase = Df(1)ase-1. (D–E) ase = Df(1)ase-1. SoxN = SoxNNC14/Df(2L)Exel7040. wor = wor4/Df(2L)ED1054. hb = hbP1, hbFB. Kr = Kr1, KrCD. nub, pdm2 = Df(2L)ED773. Ase, Asense; Dpn, Deadpan; EF, Early Factor; Hb, Hunchback; Kr, Kruppel; NB, neuroblast; Nub, Nubbin; Pdm, POU domain; PH3, phosphorylated Ser10 on Histone-H3; Pros, Prospero; St, Stage; SoxN, SoxNeuro; Wor, Worniou. (PDF) [file pbio.3000163.s002.pdf]

# Supplemental Figure 3

## Combinatorial misexpression of 6 Early Factors

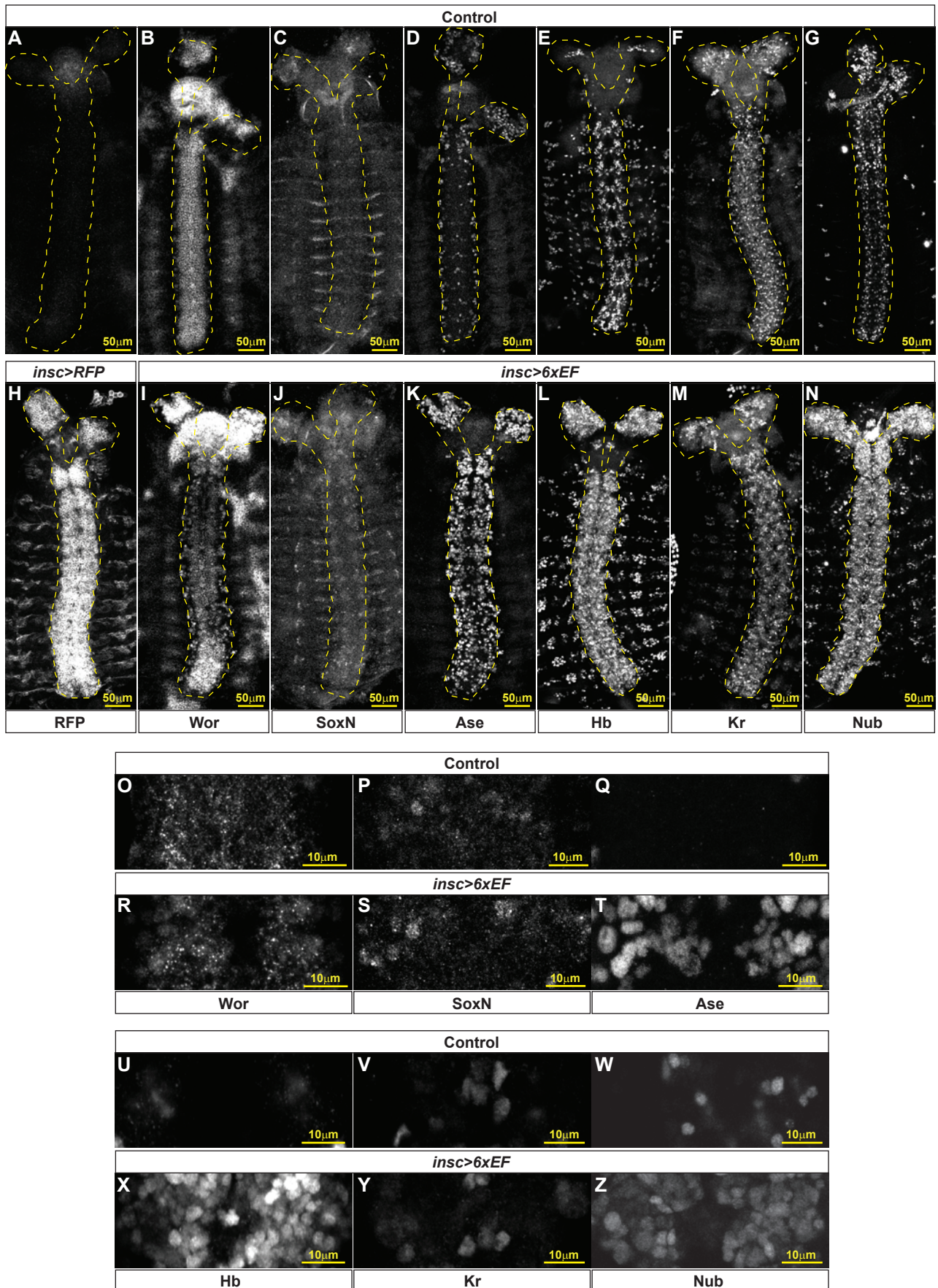

Supplement: S3 Fig — (A–N) Embryonic fillets, showing expression of RFP and the six EFs, in control and insc-Gal4/UAS embryos, at St15. (H) RFP expression shows that insc-Gal4 drives expression in the entire CNS. (B–G, I–N) insc-Gal4/UAS-6xEF embryos reveal elevated expression of all six EFs in the CNS. (O–Z) Expression of the six EFs in control and insc-Gal4/UAS-6xEF embryos, thoracic segment T2, at St15, NB layer (identified by Dpn staining). insc-Gal4 drives elevated EF expression in NBs. Genotypes: (A–G, O–Q, U–W) OregonR. (H–N, R–T, X–Z) insc-Gal4/UAS-6xEF. CNS, central nervous system; EF, Early Factor; insc-Gal4, XXX; NB, neuroblast; RFP, Red Fluorescent Protein; St, Stage; UAS, upstream activating sequence. (PDF) [file pbio.3000163.s003.pdf]

# Supplemental Figure 4

Early Factor co-misexpression overrides the Type I->0 switch and NB exit

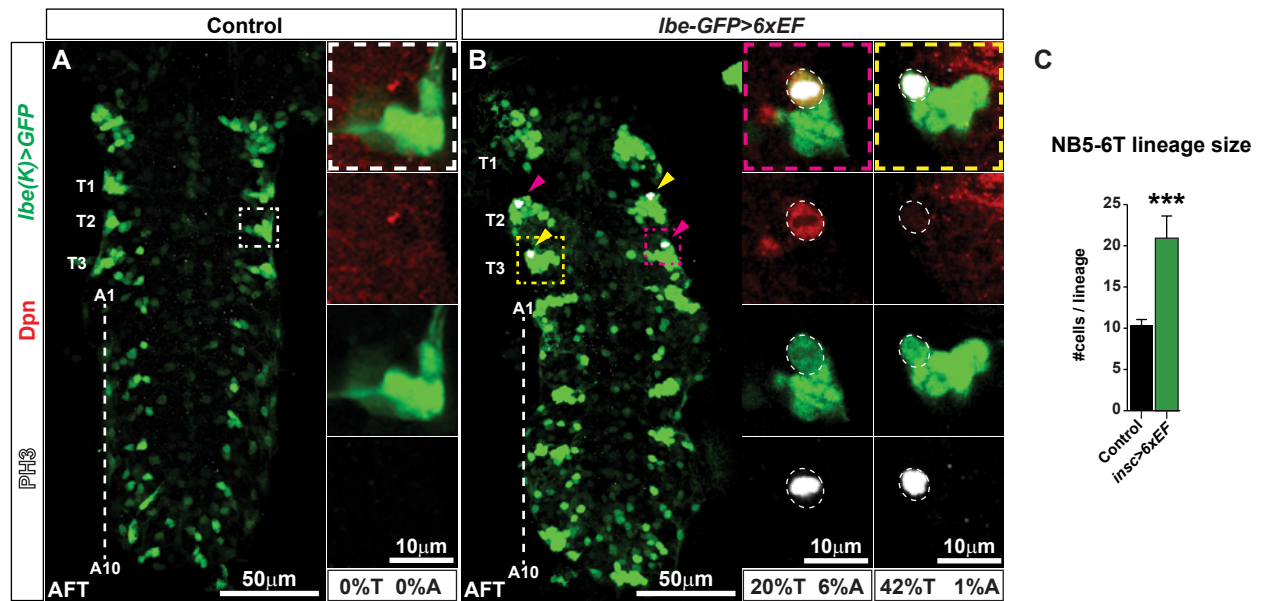

Supplement: S4 Fig — (A–B) NB5-6T lineages at stage AFT in control and lbe(K)-Gal4/UAS-6xEF dissected CNSs. Boxed regions are magnified to the right. In control, no divisions are observed in NB5-6, neither in T nor A segments. In 6xEF co-misexpression, dividing NBs and daughter cells can be observed in both T and A segments, and the lineage is larger. (C) Quantification of the number of cells in NB5-6T at stage AFT (*p ≤ 0.05, **p ≤ 0.01, ***p ≤ 0.001, Student two-tailed t test; n = 40 lineages; ±SD). The numerical data underlying this figure are included in S1 Data. Genotypes: (A) lbe(K)-Gal4, UAS-nls-myc-EGFP/+. (B) lbe(K)-Gal4, UAS-nls-myc-EGFP/UAS-6xEF. A, abdominal; AFT, air-filled trachea; EF, Early Factor; EGFP, Enhanced Green Fluorescent Protein; Gal4, Galactose4; lbe(K), ladybird early gene fragment K; myc, C-myc epitope tag; NB, neuroblast; nls, nuclear localization signal; T, thoracic; UAS, upstream activating sequence. (PDF) [file pbio.3000163.s004.pdf]

## Supplemental Figure 5

esc mutants display loss of H3K27m3 and anterior Hox expression

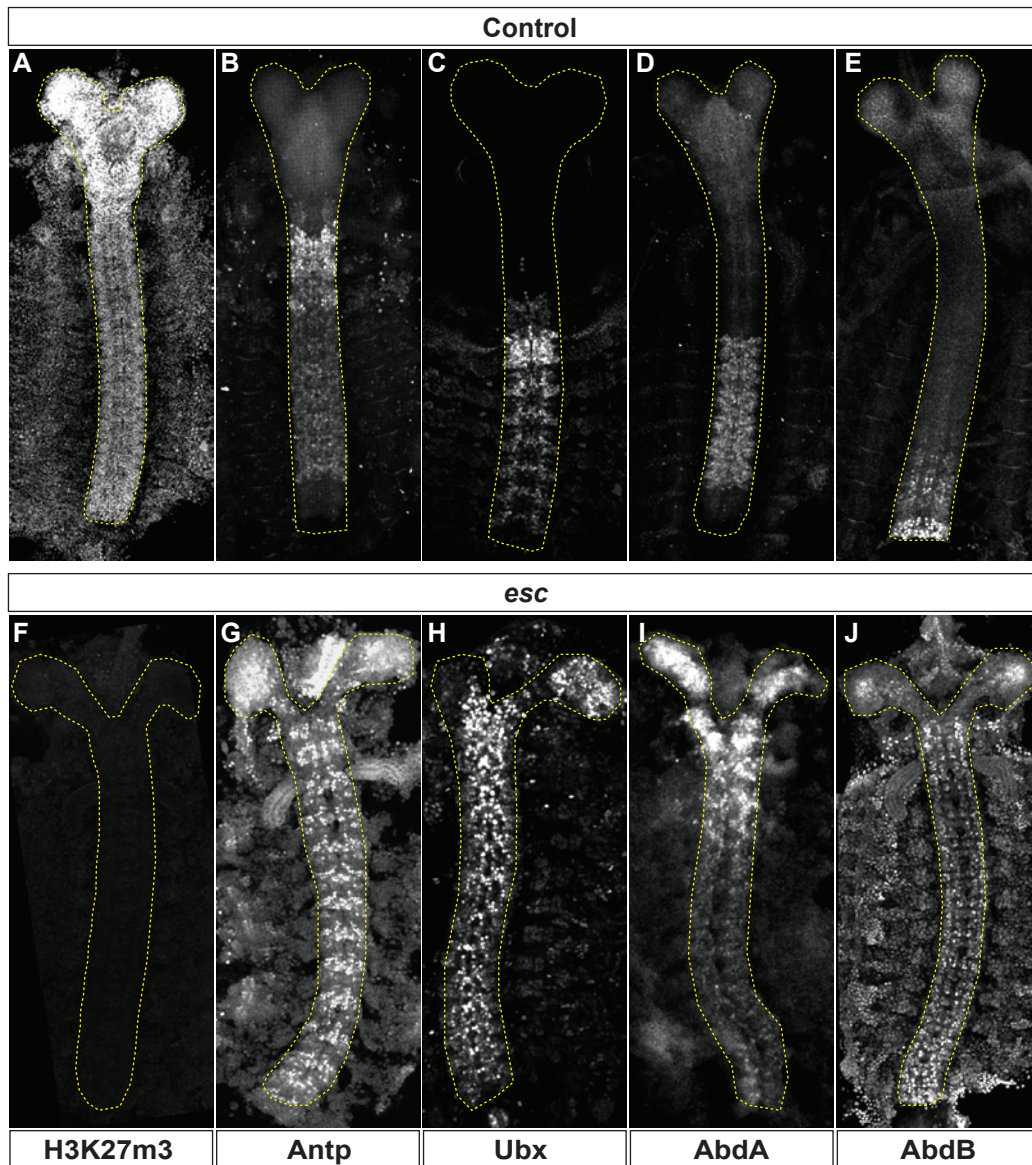

Supplement: S5 Fig — (A–J) Expression of H3K27me3, Antp, Ubx, Abd-A, and Abd-B in control and esc maternal/zygotic mutants, St15. Dashed lines outline the CNS. In esc mutants, there are nondetectable levels of H3K27me3, and all four Hox factors are expressed along the entire A–P axis, including in the brain. The numerical data underlying this figure are included in S1 Data. Genotypes: (A–E) OregonR. (F-J) esc5 or esc21 over escDf (Df(2L)Exel6030). abd-A, abdominal-A; Abd-B, Abdominal-B; Antp, Antennapedia; A–P, anterior–posterior; CNS, central nervous system; esc, extra sex combs; Hox, Homeobox; H3K27me3, Histone 3 K27 trimethylation; PRC2, Polycomb Repressor Complex 2; St, Stage; Ubx, Ultrabithorax. (PDF) [file pbio.3000163.s005.pdf]
